# Safety and efficacy of RFA versus MWA for T1a renal cell carcinoma: a propensity score analysis

**DOI:** 10.1007/s00330-022-09110-w

**Published:** 2022-09-06

**Authors:** Brigit M. Aarts, Fernando M. Gomez, Marta Lopez-Yurda, Rob F. M. Bevers, Joris Herndriks, Regina G. H. Beets-Tan, Axel Bex, Elisabeth G. Klompenhouwer, Rutger W. van der Meer

**Affiliations:** 1 Department of Radiology, The Netherlands Cancer Institute, Plesmanlaan 121, 1066 CX Amsterdam, The Netherlands; 2 Department of Radiology, Amsterdam University Medical Center Location VU, De Boelelaan 1117, 1081 HV Amsterdam, The Netherlands; 3 Department of Interventional Radiology, Hospital Clinic Universitari de Barcelona, Carrer de Villarroel 170, 08036 Barcelona, Spain; 4 Department of Biometrics, The Netherlands Cancer Institute, Plesmanlaan 121, 1066 CX Amsterdam, The Netherlands; 5 Department of Urology, Leiden University Medical Center, Albinusdreef 2, 2333 ZA Leiden, The Netherlands; 6 Department of Radiology, Catharina Hospital, Michelangelolaan 2, 5623 EJ Eindhoven, The Netherlands; 7 GROW School for Oncology and Developmental Biology, Maastricht University Medical Center, P.O. Box 5800, 6202 AZ Maastricht, The Netherlands; 8 Department of Urology, The Netherlands Cancer Institute, Plesmanlaan 121, 1066 CX Amsterdam, The Netherlands; 9 Specialist Center for Kidney Cancer, Royal Free London NHS Foundation Trust and UCL Division of Surgery and Interventional Science, Pond Street, London, NW3 2QG UK; 10 Department of Radiology, Leiden University Medical Center, Albinusdreef 2, 2333 ZA Leiden, The Netherlands; 11 Department of Radiology, Leiden University Medical Center, Albinusdreef 2, PO Box 9600, 2300 RC Leiden, The Netherlands

**Keywords:** Renal cell carcinoma, Ablation, Radiology, interventional, Propensity score, neoplasm

## Abstract

**Objectives:**

Percutaneous radiofrequency ablation (RFA) is stated as a treatment option for renal cell carcinoma (RCC) smaller than 4 cm (T1a). Microwave ablation (MWA) is a newer technique and is still considered experimental in some guidelines. The objective of this study was to compare the safety and efficacy of RFA and MWA for the treatment of RCC.

**Methods:**

Patients with T1a RCC treated by RFA or MWA in two referral centers were retrospectively analyzed. Patient records were evaluated to generate mRENAL nephrometry scores. Local tumor progression (LTP) was considered when new (recurrence) or residual tumor enhancement within/adjacent to the ablation zone was objectified. Differences in LTP-free interval (residual + recurrence) between ablation techniques were assessed with Cox proportional hazards models and propensity score (PS) methods.

**Results:**

In 164 patients, 87 RFAs and 101 MWAs were performed for 188 RCCs. The primary efficacy rate was 92% (80/87) for RFA and 91% (92/101) for MWA. Sixteen patients had residual disease (RFA (*n* = 7), MWA (*n* = 9)) and 9 patients developed recurrence (RFA (*n* = 7), MWA (*n* = 2)). LTP-free interval was significantly worse for higher mRENAL nephrometry scores. No difference in LTP-free interval was found between RFA and MWA in a model with inverse probability weighting using PS (HR = 0.99, 95% CI 0.35–2.81, *p* = 0.98) and in a PS-matched dataset with 110 observations (HR = 0.82, 95% CI 0.16–4.31, *p* = 0.82). Twenty-eight (14.9%) complications (Clavien-Dindo grade I–IVa) occurred (RFA *n* = 14, MWA *n* = 14).

**Conclusion:**

Primary efficacy for ablation of RCC is high for both RFA and MWA. No differences in efficacy and safety were observed between RFA and MWA.

**Key Points:**

*• Both RFA and MWA are safe and effective ablation techniques in the treatment of T1a renal cell carcinomas.*

*• High modified RENAL nephrometry scores are associated with shorter local tumor progression-free interval.*

*• MWA can be used as heat-based ablation technique comparable to RFA for the treatment of T1a renal cell carcinomas.*

## Introduction

Percutaneous ablation techniques are stated as a treatment option in patients with non-metastatic renal cell carcinoma (RCC) smaller than 4 cm (T1a) in urological and oncological guidelines [1, 2]. Percutaneous ablation techniques provide a minimally invasive treatment option associated with a lower morbidity rate and less decline in kidney function compared to partial nephrectomy [3]. Radiofrequency ablation (RFA) and cryoablation are the most reported ablation techniques in the kidney [4, 5]. Microwave ablation (MWA) is a relatively newer ablation technique with fewer reports available with long-term follow-up data and therefore still considered experimental/nonstandard in some guidelines [1, 2, 6, 7].

Both RFA and MWA are heat-based ablation techniques with differently applied energies. RFA is based on creating an alternating electric field around the RFA probe thereby agitating ions surrounding the probe. Ionic agitation results in frictional heat within several millimeters around the probe. Thermal conduction into more peripheral areas around the electrode creates the final ablation zone. Therefore, RFA is highly dependent on tissue properties of thermal and electric conductivity. In highly perfused renal tissue, RFA is limited by the heat sink effect of surrounding structures and by the cauterization of tissue at high temperatures [4, 8, 9]. For MWA, electromagnetic energy is applied causing heating in a volume around the MW antenna without the need of an electrically conductive path. MWA produces faster formation of the ablation zone with higher temperatures and thereby is less susceptible to heat sink compared to RFA [10, 11]. Efficacy rates of RFA for treatment of renal T1a tumors range from 73.5 to 97% with best results in lesions smaller than 3 cm [12–15]. Primary efficacy rates of MWA for T1a tumors range from 84 up to 100%, with again inferior results in lesions extending 3 cm [16–18].

To our knowledge, no significant differences between RFA and MWA regarding oncological and procedural outcomes are reported but literature comparing these two ablation techniques is sparse [12, 19, 20]. Therefore, the purpose of this study was to retrospectively compare the safety and efficacy of RFA and MWA for the treatment of T1a RCC by means of propensity score matching. In addition, we identified predictors for efficacy rates.

## Methods

### Study population

A retrospective analysis was performed in two referral institutes of patients treated for T1a RCC from 2004 to 2018. The institutional review board of both institutes approved this study and waived the requirement for informed consent.

Patients treated with RFA or MWA for a histologically proven RCC were included. Patients with tumors up to 4 cm and at least 10 months of follow-up by CT were included. The decision for percutaneous ablation was made by the multidisciplinary tumor board including an urologist, an oncologist, and an interventional radiologist. The decision between RFA and MWA as percutaneous ablation techniques was based on the physician’s preference and expertise. An overnight stay after the ablative procedure was standard practice.

### Procedures

All procedures were performed in an equipped CT suite (using either a Somatom Sensation open, Siemens Healthcare, a Philips AV (single slice), Philips Healthcare, or a Toshiba Aquillion (4, 16, or 64), Canon Medical Systems). For RFA, the Cool-tip™ RF Ablation System E Series (Medtronic) was used in both centers and the RITA 1500 (Angiodynamics) was used in the Leiden University Medical Center as well. In the Leiden University Medical Center, the AMICA MWA System (HS Hospital Service) was used. In the Netherlands Cancer Institute, the Evident MW system (Medtronic) and later the Emprint MW system (Medtronic) were used. For the positioning of the RFA or MWA needles, ultrasound and/or non-contrast-enhanced CT-guided needle placement was used. Iso-attenuating masses were identified by their contours outside the renal parenchyma or by surrounding structures. Only sporadically a contrast-enhanced CT scan was performed before ablation to identify the lesion. Hydrodissection and/or ureter perfusion was used for the protection of nearby structures when necessary. During RFA, single or multiple probes were used depending on tumor size and the operator’s discretion. RFA time ranged from 10 to 23 min. During RFA, power was automatically adjusted based on impedance or temperature changes to optimize energy delivery. For MWA a power of 45–100 watts for 2–10 min was used. When probe placement on a single location was not sufficient to cover the entire tumor, repeated probe placement was performed.

After ablation, a contrast-enhanced CT was performed to confirm a sufficient ablation zone around the tumor. When an insufficient ablation zone was observed, additional ablation was performed immediately.

### Follow-up

Each patient entered the institutional follow-up scheme at the outpatient clinic of the interventional radiologist or urologist. In the Leiden University Medical Center, the first multiphase CT scan was performed after 2 months and after that semi-annually within the first 3 years. In the Netherlands Cancer Institute, multiphase CT scans after 1, 3, 6, 9, and 12 months were performed. Patients were offered to continue follow-up at their primary center after 1 year of local control. One multiphase CT scan each year during the first 5 years after the ablation was advised to the referring urologist at the primary center.

The date of the last available multiphase CT was considered as the end of follow-up.

### Study endpoints

Tumor characteristics were retrospectively assessed to identify predictors of efficacy. The modified RENAL nephrometry score (which has been shown to better predict the success of ablation than the original RENAL nephrotomy score used for characterizing the complexity of surgical resection [21]) was calculated. For calculating the mRENAL nephrotomy score the tumor radius, centrality (exophytic/endophytic), nearness to the collecting system or sinus, and location relative to polar lines were assessed [21, 22]. Primary efficacy was achieved when no tumor enhancement occurred on the follow-up CT scan (2–3 months after ablation) [23]. Local tumor progression (LTP) was considered when a new nodule enhancement (recurrence) or residual tumor enhancement (residual) was objectified in, or in direct contact with, the ablation zone. Time to LTP (LTP-free interval) was defined as the time from ablation until a recurrence or residual disease. Adverse events post-ablation were classified according to the Clavien-Dindo classification [24]. Change in kidney function was measured by extracting the first available estimated glomerular filtration rate (eGFR) after 1–3 months from the eGFR before ablation (according to the center’s guidelines).

### Statistical analysis

The Kaplan-Meier method was used to generate survival curves and comparisons between ablation techniques were performed with the log-rank test. Patients without an LTP before death or end of follow-up were censored. Univariable and multivariable Cox proportional hazards regression analyses, stratified by center, were performed. Given the relatively small number of events, the selection of covariates for a multivariable analysis was based on their significance in the univariable analysis and their clinical importance. Clinical variables used in the determination of the mRENAL nephrometry score were not introduced as covariates together with the latter. The small number of events does not allow an adequate adjustment for all relevant baseline variables in a multivariable model. As a more flexible alternative, a propensity score model was estimated, and stabilized inverse probability weights (IPW) were calculated in order to estimate a weighted Cox proportional hazards model. In addition, one-to-one propensity score matching of RFA to MWA patients was done using greedy nearest neighbor matching with caliper 0.1. Previous renal tumor, ASA score, pathology, size, mRENAL nephrometry score, location, and other clinical and demographic characteristics were identified as candidate variables for the propensity score model. Different functional forms and interactions between variables were considered. To account for multiple lesions within patients in all analyses, and for the matched nature of the data in the propensity score matching, standard errors were calculated using the jackknife sandwich estimator.

The proportional hazard assumption was assessed by scaled Schoenfeld residual plots over time. Cut-off points for grouped continuous variables are in alignment with previous studies and were also evaluated by martingale residual plots. Median follow-up was calculated from the time of ablation using the reverse Kaplan-Meier method.

Statistical comparison was done using Pearson’s chi-square test or Fisher’s exact test for categorical variables, and Mann-Whitney’s U-test for continuous variables. All statistical tests were two-tailed. All analyses were performed using R (version 3.5) and SAS (Version 9.4).

## Results

### Patient and tumor characteristics

**Fig. 1:**
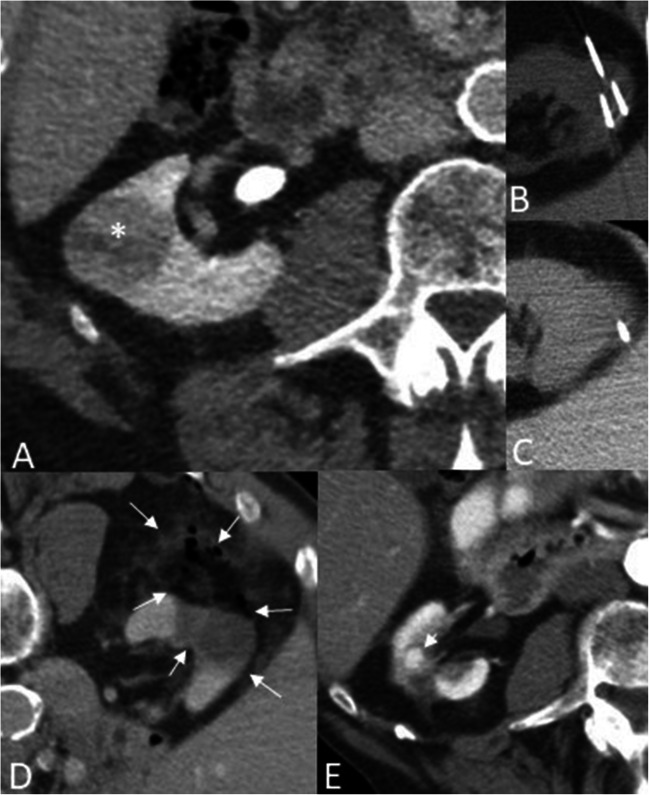
A RCC treated by RFA. A pretreatment contrast-enhanced CT image with a RCC in the right kidney is shown (* in panel **A**). In panel **B** and **C** needle placement in consecutive images during RFA is shown. Three RFA needles are placed through the tumor. Note that the RFA procedure was performed with the patient in a prone position. Panel **D** shows an image directly after ablation. The ablation zone is marked by arrows. Ablation was considered successful; however, 1 year later, a local recurrence was noted (arrow in panel **E**, patient in supine position). The recurrence was successfully treated with a second RFA procedure

Between 2004 and 2018, a total of 337 renal masses were treated by RFA or MWA. Of these, 149 were excluded because of a benign, non-diagnostic, or no pathology result. As a result, 188 histologically proven RCCs in 164 unique patients treated in 167 treatments by means of RFA (*n* = 87) or MWA (*n* = 101), were included in the analyses. Pretreatment patient and tumor characteristics are shown in Table 1. In Fig. 1, CT scans of a treated RCC are shown.

**Table 1:** Baseline demographic and clinical characteristics

	Unmatched cohort				Matched cohort			
	RFA (*N* = 87)	MWA (*N* = 101)	Total (*N* = 188)	*p* value	RFA (*N* = 55)	MWA (*N* = 55)	Total (*N* = 110)	*p* value
Gender				0.755				1.000
Female	29 (33%)	31 (31%)	60 (32%)		16 (29%)	17 (31%)	33 (30%)	
Male	58 (67%)	70 (69%)	128 (68%)		39 (71%)	38 (69%)	77 (70%)	
Median age at ablation (years, Q1–Q3)	65 (59–72)	68 (60–73)	67 (60–73)	0.18	65 (60–72)	65 (59–72)	65 (59–72)	0.751
ASA-score				0.025				0.700
Missing	4	2	6					
0–1	12 (14.5%)	19 (19.2%)	31 (17.0%)		9 (16%)	9 (16%)	18 (16%)	
2	63 (75.9%)	58 (58.6%)	121 (66.5%)		39 (71%)	35 (64%)	74 (67%)	
3–4	8 (9.6%)	22 (22.2%)	30 (16.5%)		7 (13%)	11 (20%)	18 (16%)	
Tumor type				0.016				
Chromophobe	6 (7%)	4 (4%)	10 (5%)		2 (4%)	3 (6%)	5 (5%)	0.999
Clear cell	54 (62%)	55 (55%)	109 (58%)		35 (64%)	35 (64%)	70 (64%)	
Eosinophilic	2 (2%)	0 (0.0%)	2 (1%)		1 (2%)	0 (0%)	1 (1%)	
Papillair	24 (28%)	30 (30%)	54 (29%)		16 (29%)	16 (29%)	32 (29%)	
RCC but undefined	1 (1%)	12 (11%)	13 (7%)		1 (2%)	1 (2%)	2 (2%)	
Tumor side				0.131				0.999
Right	42 (48%)	57 (56%)	99 (53%)		30 (55%)	30 (54%)	60 (54%)	
Left	42 (48%)	44 (44%)	86 (46%)		25 (45%)	25 (46%)	50 (46%)	
Renal transplant	3 (4%)	0 (0.0%)	3 (1%)		–––	––	––	
Median tumor size (cm, Q1–Q3)	2.6 (1.8–3.0)	2.6 (2.1–3.2)	2.6 (2.0–3.2)	0.165	2.6 (1.9–2.9)	2.4 (1.9–3.2)	2.5 (1.9–3.1)	0.928
Tumor size, grouped -				0.263				0.512
< = 3 cm	65 (75%)	67 (66%)	132 (70%)		43 (78%)	39 (70.9%)	82 (74.5%)	
> 3 cm	22 (25%)	34 (34%)	56 (30%)		12 (22%)	16 (29.1%)	28 (25.5%)	
Centrality				0.544				0.771
< 50% exophytic	26 (29%)	23 (23%)	49 (26%)		15 (27%)	18 (33%)	33 (30%)	
> 50% exophytic	37 (43%)	48 (48%)	85 (45%)		23 (42%)	23 (42%)	46 (42%)	
Endophytic	24 (28%)	30 (29%)	54 (29%)		17 (31%)	14 (25%)	31 (28%)	
Nearness to the collecting system				< 0.001				0.192
> 7 mm	29 (34%)	67 (66%)	96 (51%)		22 (40%)	31 (56%)	53 (48%)	
4–7 mm	14 (16%)	12 (12%)	26 (14%)		11 (20%)	6 (11%)	17 (16%)	
< 4 mm	44 (50%)	22 (22%)	66 (35%)		22 (40%)	18 (33%)	53 (48%)	
RENAL L				0.002				0.683
Missing	0	1	1					
Entirely above	39 (45%)	61 (61%)	100 (54%)		33 (60%)	28 (51%)	61 (56%)	
Crosses 1 polar line	23 (26%)	29 (29%)	52 (28%)		15 (27%)	18 (33%)	33 (30%)	
Crosses 2 polar lines/50% across axial	25 (29%)	10 (10%)	35 (20%)		7 (13%)	9 (16%)	16 (14%)	
Median mRENAL score (Q1–Q3)	7 (6–8)	6 (5–8)	6 (5–8)	< 0.001	7.0 (5.0–8.0)	6.0 (5.0–8.0)	6.0 (5.0–8.0)	0.534

*RFA* radiofrequency ablation; *MWA* microwave ablation; *ASA* American Society of Anesthesiologists; *RCC* renal cell carcinoma

In the RFA group, tumors were significantly closer to the collecting system (< 4 mm; RFA 50% vs MWA 22%, *p* < 0.001) and the polar lines (crosses 2 polar lines; RFA 29% vs MWA 10%, *p = *0.003) reflecting in a higher mRENAL nephrometry score (RFA median = 7 vs MWA median = 6, *p* < 0.001).

### Treatment characteristics

Ablations were performed under CT guidance in most of the procedures or in a hybrid setting consisting of a combination of ultrasound and CT. Epidural anesthesia was used in 36% of the RFA procedures and in 88% of the MWA procedures (*p* < 0.001), mainly in The Netherlands Cancer Institute where epidural anesthesia was standard practice for renal ablative therapies. In the other institutions, general anesthesia was mostly used.

### Oncological outcome

Median follow-up was 4.26 (interquartile range 1.95–5.51) years in the RFA group and 1.62 (interquartile range 1.08–2.67) years in the MWA group. Primary efficacy rates yielded 92% (80/87 tumors) for the RFA group and 91% (92/101 tumors) for the MWA group. Sixteen patients showed residual disease on the first follow-up scan after 3 months (RFA (*n* = 7) and MWA (*n* = 9)). Nine patients developed recurrence after RFA (*n* = 7) and MWA (*n* = 2). In one patient, local progression was suspected on CT and this patient was treated by surgery, but no viable tumor cells were observed at the pathologic specimen.

Seventeen of the 25 residual/recurrent tumors were treated by ablation in a repeated procedure. The other 9 tumors were treated by means of surgery (*n* = 5), active surveillance (*n = *3), and stereotactic body radiation (*n* = 1). The second ablation was successful in 14/17 tumors. Three tumors needed a third ablation, after which 1 patient (treated with MWA) needed surgery for completion therapy. This patient had a complete endophytic tumor with a close relation to the renal artery.

No difference was observed between RFA and MWA for change in kidney function using the estimated glomerular filtration rate (eGFR) (Table 2). Eight patients developed metastases (5 after RFA and 3 after MWA) during follow-up.

**Table 2:** Procedural characteristics and post-procedural outcomes

	Unmatched cohort			Matched cohort		
	MWA (*N* = 101)	RFA (*N* = 87)	Total (*N* = 188)	MWA (*N* = 55)	RFA (*N* = 55)	Total (*N* = 110)
Anesthesia						
Epidural	89 (88%)	31 (36%)	120 (64%)	45 (81.8%)	25 (45.5%)	70 (63.6%)
General	12 (12%)	56 (64%)	68 (36%)	10 (18.2%)	30 (54.5%)	40 (36.4%)
Median eGFR pre ablation (Q1–Q3)	72 (56–82)	63 (61–87)	69 (59–83)	71 (57–78)	66 (61–87)	68 (61–80)
Median eGFR post ablation (Q1–Q3)	67 (49–77)	61 (53–79)	64 (52–78)	66 (48–76)	64 (58–80)	65 (53–76)
Median eGFR change (Q1–Q3)	−5 (–9–0)	−3 (–11–0)	−4 (–10–0)	−5 (–9–0)	−3 (–11–0)	−3 (–11–0)
Complication grade^a^						
Grade I	8 (8%)	6 (7%)	14 (7%)	4 (7%)	4 (7%)	8 (7%)
Grade II	5 (5%)	6 (7%)	11 (6%)	5 (9%)	2 (4%)	7 (6%)
Grade IIIa	0 (0.0%)	1 (1%)	1 (1%)	--	--	--
Grade IIIb	0 (0.0%)	1 (1%)	1 (1%)	0 (0.0%)	1 (2%)	1 (1%)
Grade IVa	1 (1%)	1 (1%)	2 (1%)	0 (0.0%)	1 (2%)	1 (1%)
Total grade I–IVa	14 (14%)	15 (17%)	29 (15%)	9 (16%)	8 (15%)	17 (15%)

^a^Over total number of patients in cohort

*MWA* Microwave ablation; *RFA* Radiofrequency ablation; *eGFR* Glomerular filtration rate

### Local tumor progression

In total, 25 patients developed LTP (RFA *n* = 14, MWA *n* = 11). Time to LTP ranged from 1 month to 7.8 years for RFA and 1 week to 5.2 years for MWA.

**Fig. 2:**
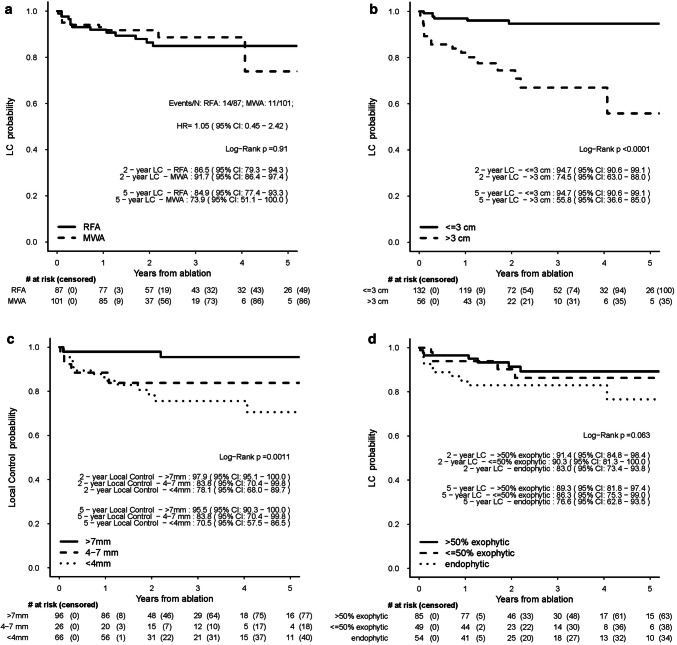
**a** Local control (LC) from date of ablation to tumor progression for the RFA and MWA group. **b** Local control (LC) from date of ablation to tumor progression divided by tumor size larger than 3 cm and smaller than 3 cm (pooled data of RFA and MWA groups). **c** Local control (LC) from date of ablation to tumor progression for distance to the collection system (> 7 mm, 4–7 mm, < 4 mm, pooled data of RFA and MWA groups). **d** Local control (LC) from date of ablation to tumor progression for exophytic or endophytic tumors (pooled data of RFA and MWA groups)

There was no difference in the LTP-free interval between RFA and MWA in a univariable Cox regression model (Fig. 2A). Results from a multivariable Cox regression analysis (see Table 3) adjusting for mRENAL nephrometry score and stratified by center did not show a significant difference between ablation techniques (HR = 0.87, 95% CI 0.33–2.28, *p* = 0.78).

**Table 3:** Cox proportional hazards regression results for local control

				Univariable		Multivariable^a^		IPW		PS matching^b^	
		Events	N	HR (95% CI)	*p*	HR (95% CI)	*p*	HR (95% CI)	*p*	HR (95% CI)	*p*
Ablation technique	MWA	11	101	1.05 (0.43–2.54)	0.910	0.93 (0.37–2.35)	0.88	0.99 (0.35–2.81)	0.98	0.82 (0.16–4.31)	0.82
	RFA	14	87	1 (ref.)							
mRENAL score		25	187^c^	1.61 (1.32–1.97)	< 0.001	1.60 (1.34–1.92)	< 0.001				
Tumor size (cm)	per unit	25	188	4.64 (2.30–9.35)	< 0.001						
Tumor size (grouped)	> 3 cm	17	56	6.78 (2.90–15.86)	< 0.001						
	< = 3 cm	8	132	1 (ref.)							
Nearness collecting system	< 4 mm	16	66	6.16 (2.05–18.49)	0.001						
	4–7 mm	5	26	4.89 (1.29–18.53)	0.020						
	> 7 mm	4	96	1 (ref.)							
Location	< 50% exophytic	5	49	1.20 (0.39–3.69)	0.745						
	Endophytic	12	54	2.65 (1.10–6.42)	0.030						
	> 50% exophytic	8	85	1 (ref.)							

*IPW* inverse probability weighting; *PS* propensity score; *HR* hazard ratio; *CI* confidence interval; *MWA* microwave ablation; *RFA* radiofrequency ablation

^a^Stratified by center

^b^Based on 55 matched pairs

^c^In one patient calculation of the mRENAL score was not possible due to the anatomic variation of a horseshoe kidney

Univariable Cox regression analyses showed that increasing mRENAL nephrometry score, size (> 3 cm), nearness to the collecting system (< 7 mm), and endophytic location were associated with a worse LTP-free interval (Fig. 2B–D and Table 3). Similar conclusions were obtained in subgroup analyses for RFA and MWA patients separately, though for the MWA subgroup nearness to the collecting system 4–7 mm appeared to have poorer local control than < 7 mm. Given the small numbers in each of these subgroups and the similarities with overall results, these analyses are not displayed.

No evidence was found in a separate model adjusting for size and nearness of the collecting system (HR = 0.76, 95% CI 0.31–1.88, *p* = 0.55). In this model, increasing size remained associated with poorer LTP-free interval (HR = 3.81, 95% CI 1.53–9.44, *p* = 0.004) as was the case for nearness to the collecting system 4–7 mm and < 4 mm (HR > 5 in both cases) (Table 3). Results from a Cox regression model with inverse probability weighting using the propensity score showed similar results (HR = 0.99, 95% 0.35–2.81, *p* = 0.98). For a propensity score-matched dataset using one-to-one greedy nearest neighbor matching without replacement, conclusions were similar (HR = 0.82, 95% CI 0.16–4.31, *p* = 0.82). Matching induced balance in all baseline covariates (see Table 1), though it led to 41% of the data being unmatched (55 matched pairs) and thus a loss of precision and generalizability.

Given the difference in median follow-up time for both ablation techniques (4.26 years for RFA [interquartile range 1.95–5.51] and 1.62 years for MWA [interquartile range 1.08—2.67]), a sensitivity analysis was performed by truncating time to LTP at 6.52 years (the maximum follow-up in the arm with the shortest follow-up [MWA]) in multivariable analyses. No differences in LTP-free interval were found (results not shown).

### Adverse events

After the 188 primary ablations, 28 (14.9%) complications occurred after 14 RFA procedures and 14 MWA procedures (Table 2). There were no peri-procedural deaths. There were four major adverse events (3%), 3 after RFA (1.6%) and 1 after MWA (0.53%). Damage to the pyelocaliceal system led to a significant stenosis in all four patients. In two patients, this stenosis caused total failure of the ablated kidney (1 RFA, 1 MWA) (grade IVa). In one patient, a fistula between a renal calix and the skin developed after RFA, complicated by abscess formation and sepsis for which the patient was admitted to the ICU (grade IIIb). The fourth patient needed double J placement (grade IIIa) for the treatment of the stenosis. Twenty-four (12.8%) minor adverse events occurred, 10 (5.3%) after RFA and 13 (7.0%) after MWA, and consisted of pain, hematoma, hematuria, or pneumothorax in most of the cases.

## Discussion

In this multicenter study, we showed equal results for RFA and MWA in the treatment of T1a RCC using Cox regression analyses and propensity score methods. No significant differences were found for primary efficacy, change in eGFR, and complications.

Even though no report to date has shown inferiority of MWA compared to RFA or cryoablation, MWA is considered an experimental ablation technique in the European Association of Urology RCC guidelines [1]. Yu et al even reported equal results between MWA and laparoscopic partial nephrectomy (which is considered treatment of preference by the European Association of Urology) in terms of LTP, overall survival, and cancer-specific survival in a recent report. Patients in the MWA group showed a significantly higher Charlson comorbidity index, even after propensity score matching, indicating a bias in patient selection [25]. This raises the question if ablative therapies should not be considered in all patients with T1a RCC given its lower risk profile compared to partial nephrectomy.

In our study, 25 tumors showed LTP during follow-up. Multivariable Cox regression analyses, adjusting for mRENAL nephrometry score, showed no significant difference between RFA and MWA in LTP-free interval. With propensity score methods, no significant differences were observed either, though results are hampered by the relatively low number of events available, reflected in wide confidence intervals.

Our results are in line with the current literature showing no difference between RFA and MWA [12, 19, 20] for treating T1a RCC. In addition to the current literature, propensity score methods were used to induce balance in important clinical factors between groups, and to overcome limitations in the number of events per parameter in the multivariable Cox regression model. To specify, before the matching process, the RFA group contained significantly more tumors with a closer distance to the collecting system and the polar lines producing a higher mRENAL nephrometry score. Since these parameters influence LTP, a comparison would be biased. After the propensity matching process, the groups were equalized containing an even number of patients with these factors. Only 110 patients (61%) remained after matching with a consequent lower power and loss of precision and generalizability, therefore caution should be made for the interpretation of these results.

Multivariable Cox regression showed a significant association for mRENAL nephrometry score with LTP. In the literature, RFA and MWA both show limitations for treating larger centrally localized tumors. Abboud et al compared RFA to MWA and showed an association between the RENAL nephrometry score and RFA, but not for MWA [19], but only 17 lesions were treated with MWA in this study. Zhou et al, who compared 44 MWA with 347 RFAs and 46 cryoablations, did show a significant association between the RENAL nephrometry score and LTP for all patients [20]. Since MWA provides larger ablation zones with higher temperatures [26], size and central location would be less of an issue, only our results and literature suggest counter-wise.

This study contains some limitations. The major limitation of this study was the retrospective study design. Due to the nature of the data, follow-up in the RFA group was longer than in the MWA group that could affect LTP. Sensitivity analyses truncating time to LTP to make follow-up time between groups comparable were also performed and did not find evidence of a difference in LTP-free interval between patients treated with RFA or MWA. Furthermore, there were few events for analysis. It is unclear what level of difference in terms of cancer control and other measures between the two groups could have been detected. Finally, this study is limited by the introduction of biases due to this being an observational cohort where the ablation technique applied was based on the physician’s preference (giving rise to confounding by unmeasured factors, which cannot be properly addressed solely by statistical methodology). It is thus our recommendation to compare the ablation techniques in this manuscript in the context of a prospective randomized study, which would be able to tackle the aforementioned issues.

In conclusion, we showed high primary efficacy for both MWA and RFA in the treatment of T1a RCC. In addition, a high mRENAL nephrometry score was associated with a shorter LTP-free interval. Finally, equal success between RFA and MWA in the treatment of T1a RCC patients in terms of safety, efficacy, and LTP using propensity score methods was shown. Therefore, MWA could be used as heat-based ablation technique comparable to RFA for the treatment of T1a RCC.

## Abbreviations

eGFR: Estimated glomerular filtration rate
IPW: Inverse probability weighting
LTP: Local tumor progression
mRENAL: Modified RENAL score (radius, exophytic/endophytic centrality, nearness to collecting system or sinus, anterior/posterior, and location relative to polar lines)
MWA: Microwave ablation
PS: Propensity score
RCC: Renal cell carcinoma
RFA: Radiofrequency ablation

## Declarations

### Guarantor

The scientific guarantor of this publication is RW van der Meer.

### Statistics and biometry

One of the authors has significant statistical expertise.

### Study subjects or cohorts overlap

Some of the patients from 1 center included in this study were also part of the analysed patients in another publication (*Cardiovasc Intervent Radiol*. 2020 Jul;43(7):1025–1033. 10.1007/s00270-020-02423-7. Epub 2020 Feb 12.)

### Methodology

• retrospective

• case-control study

• multicenter study
